# (*E*)-1-[1-(4-Chloro­phen­yl)ethyl­idene]thio­semicarbazide

**DOI:** 10.1107/S1600536812029133

**Published:** 2012-07-04

**Authors:** Rafael Mendoza-Meroño, Santiago García-Granda

**Affiliations:** a Departamento de Química Física y Analítica, Facultad de Química, Universidad de Oviedo – CINN, C/ Julián Clavería, 8, 33006 Oviedo, Spain

## Abstract

In the crystal structure of the title compound, C_9_H_10_ClN_3_S, the mol­ecules form chains parallel to [001] through N—H⋯S hydrogen bonds. In addition, weak inter­molecular N—H⋯Cl hydrogen bonds connect the chains, forming a two-dimensional network parallel to (001).

## Related literature

For related compounds and their biological activity, see: Odenike *et al.* (2008 ▶); Rebolledo *et al.* (2008 ▶). For a related structure, see: Wang *et al.* (2007 ▶). For a description of the Cambridge Structural Database, see: Allen (2002 ▶).
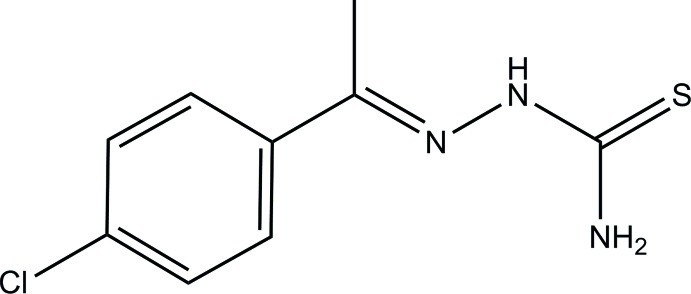


## Experimental

### 

#### Crystal data

C_9_H_10_ClN_3_S
*M* *_r_* = 227.71
Monoclinic, 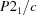

*a* = 9.2760 (2) Å
*b* = 13.9990 (3) Å
*c* = 8.3970 (2) Å
β = 97.448 (2)°
*V* = 1081.19 (4) Å^3^
*Z* = 4
Cu *K*α radiation
μ = 4.64 mm^−1^
*T* = 293 K
0.53 × 0.10 × 0.10 mm

#### Data collection

Oxford Diffraction Xcalibur (Ruby, Gemini) diffractometer
Absorption correction: multi-scan (*CrysAlis PRO*; Agilent, 2011 ▶) *T*_min_ = 0.256, *T*_max_ = 1.000
6043 measured reflections
2024 independent reflections
1807 reflections with *I* > 2σ(*I*)
*R*_int_ = 0.027

#### Refinement

*R*[*F* ^2^ > 2σ(*F* ^2^)] = 0.037
*wR*(*F* ^2^) = 0.104
*S* = 1.06
2024 reflections
167 parameters
All H-atom parameters refined
Δρ_max_ = 0.31 e Å^−3^
Δρ_min_ = −0.30 e Å^−3^

### 

Data collection: *CrysAlis CCD* (Oxford Diffraction, 2010 ▶); cell refinement: *CrysAlis CCD*; data reduction: *CrysAlis RED* (Oxford Diffraction, 2010 ▶); program(s) used to solve structure: *SIR2008* (Burla *et al.*, 2007 ▶); program(s) used to refine structure: *SHELXL97* (Sheldrick, 2008 ▶); molecular graphics: *ORTEP-3 for Windows* (Farrugia, 1997 ▶) and *Mercury* (Macrae *et al.*, 2008 ▶); software used to prepare material for publication: *WinGX* (Farrugia, 1999 ▶), *PLATON* (Spek, 2009 ▶), *PARST95* (Nardelli, 1995 ▶) and *publCIF* (Westrip, 2010 ▶).

## Supplementary Material

Crystal structure: contains datablock(s) global, I. DOI: 10.1107/S1600536812029133/kp2427sup1.cif

Structure factors: contains datablock(s) I. DOI: 10.1107/S1600536812029133/kp2427Isup2.hkl

Supplementary material file. DOI: 10.1107/S1600536812029133/kp2427Isup3.cml

Additional supplementary materials:  crystallographic information; 3D view; checkCIF report

## Figures and Tables

**Table 1:** Hydrogen-bond geometry (Å, °)

*D*—H⋯*A*	*D*—H	H⋯*A*	*D*⋯*A*	*D*—H⋯*A*
N2—H9⋯S1^i^	0.89 (3)	2.69 (3)	3.581 (2)	175 (2)
N3—H10*B*⋯S1^ii^	0.84 (3)	2.54 (3)	3.351 (2)	163 (2)
N3—H10*A*⋯Cl1^iii^	0.85 (3)	2.88 (2)	3.500 (2)	131 (2)

Symmetry codes: (i) 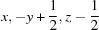; (ii) 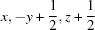; (iii) 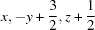.

**Fig. 1.**
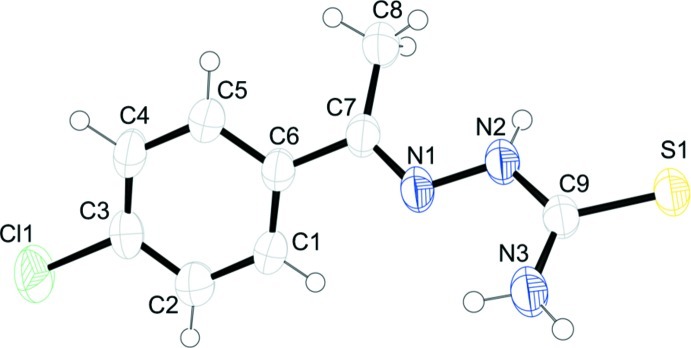
A view of the molecular structure of the title molecule showing the atom-numbering scheme. Displacement ellipsoids are drawn at the 50% probability level.

**Fig. 2.**
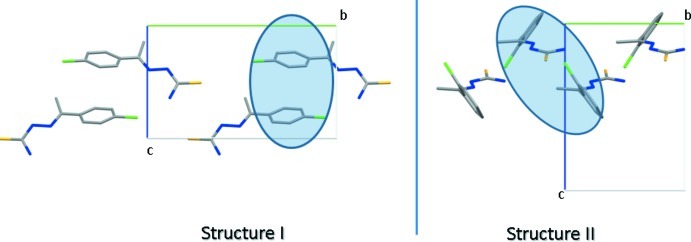
The principal differences of para-isomer (I) and ortho-isomer (II).

**Fig. 3.**
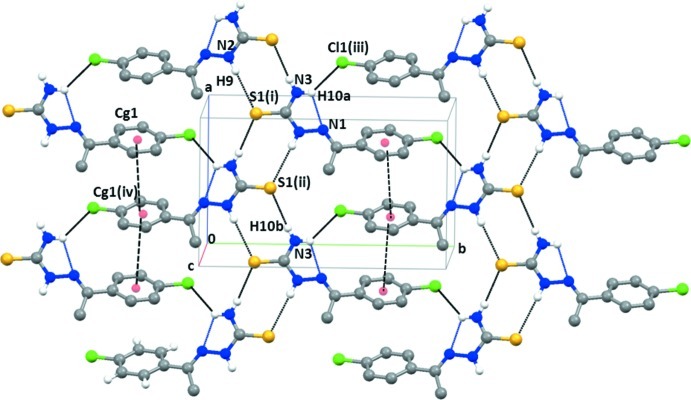
Packing diagram viewed parallel to (001). Hydrogen bonds and intermolecular interactions are indicated by dashed lines. H atoms not involved in hydrogen bonding have been omitted for clarity.

## supplementary crystallographic information

### Comment

The family of thiosemicarbazone compounds have been extensively studied due to their wide range potential in medical applications (Odenike *et al.*.,2008). Some studies with acetophenone derivates and their coordination complexes (Rebolledo *et al.* 2008) reveal that these compounds could be used as new class of *anti-trypanosomal drug* candidates. In this work we have synthesised and crystallised a new acetophenone thiosemicarbazone derivate (I).

The molecule exist in the thione form and *E*-configuration about hydrazine bond. The bond length N(1)–N(2) (1.380 (2) Å) and the dihedral angle C(7)═ N(1)—N(2)—C(9) (171.36 (2) °) are similar to those found for thiosemicarbazone systems in CSD (Allen, 2002) [selected 371 hits, average distance N—N is 1.374 Å and mean dihedral angle is 178.21 °] (Fig. 1).

The dihedral angle between chlorobenzene ring C1/C2/C3/C4/C5/C6/Cl1 (C3 atom max. deviation = 0.0098 (2) Å) and the moiety C7/N1/N2/C9/S1/N3 ( N1 atom max. deviation = 0.0955 (2) Å) is 44.25 (1)° for structure (I). The dihedral angle for the analogous, structure (II), reported by Wang (2007), in which Cl atom is in *ortho* position, is 57.48 (1)° (Fig. 2). The spatial position of para-isomer favours π–π stacking interactions of chlorobenzene rings in (I) (Fig. 3).

In the crystal packing molecules are forming chains througth N(2)—H(9)···S(1) and N(3)—H(10b)···S(1) hydrogen bonds along *a axis* (Table 1), as observed in other acetophenone thiosemicarbazone derivate, previously reported by ( Wang *et al.* 2007). Weak intermolecular N(3)—H(10a)···Cl(1) hydrogen bond and π–π stacking interactions [Cg1(C1→C6)···Cg1(iv) = 4.2142 (1) Å, offset= 31.95° and dihedral angle = 13 (2) ° for iv: x,1/2-y,-1/2+z] are present in the crystal contributing to stabilize chains.

### Experimental

A solution of 1-(4-chlorophenyl)ethanone (3.092 g 0.02 mol) and thiosemicarbazide (1.82 g, 0.02 mol) in absolute methanol (80 mL) was refluxed for 2 h in the presence of *p*-toluenesulfonic acid as catalyst, with continuous stirring. On cooling to room temperature the precipitate was filtered off, washed with copious cold methanol and dried in air. Colourless single crystals of compound (I) were obtained after recrystallisation from a solution in methanol.

### Refinement

All H atoms located at the difference Fourier maps and isotropically refined. At the end of the refinement the highest peak in the electron density was 0.310 eÅ ^-3^, while the deepest hole was -0.300 eÅ ^-3^.

### Crystal data

**Table:**

C_9_H_10_ClN_3_S	*F*(000) = 472
*M**_r_* = 227.71	*D*_x_ = 1.399 Mg m^−^^3^
Monoclinic, *P*2_1_/*c*	Cu *K*α radiation, λ = 1.54180 Å
*a* = 9.2760 (2) Å	Cell parameters from 4083 reflections
*b* = 13.9990 (3) Å	θ = 4.8–70.4°
*c* = 8.3970 (2) Å	µ = 4.64 mm^−^^1^
β = 97.448 (2)°	*T* = 293 K
*V* = 1081.19 (4) Å^3^	Needle, white
*Z* = 4	0.53 × 0.10 × 0.10 mm

### Data collection

**Table:**

Oxford Diffraction Xcalibur (Ruby, Gemini) diffractometer	2024 independent reflections
Radiation source: Enhance (Cu) X-ray Source	1807 reflections with *I* > 2σ(*I*)
Graphite monochromator	*R*_int_ = 0.027
Detector resolution: 10.2673 pixels mm^-1^	θ_max_ = 70.6°, θ_min_ = 4.8°
ω scans	*h* = −9→11
Absorption correction: multi-scan (*CrysAlis PRO*; Agilent, 2011)	*k* = −16→16
*T*_min_ = 0.256, *T*_max_ = 1.000	*l* = −10→9
6043 measured reflections	

### Refinement

**Table:**

Refinement on *F*^2^	Primary atom site location: structure-invariant direct methods
Least-squares matrix: full	Secondary atom site location: difference Fourier map
*R*[*F*^2^ > 2σ(*F*^2^)] = 0.037	Hydrogen site location: difference Fourier map
*wR*(*F*^2^) = 0.104	All H-atom parameters refined
*S* = 1.06	*w* = 1/[σ^2^(*F*_o_^2^) + (0.0575*P*)^2^ + 0.2508*P*] where *P* = (*F*_o_^2^ + 2*F*_c_^2^)/3
2024 reflections	(Δ/σ)_max_ < 0.001
167 parameters	Δρ_max_ = 0.31 e Å^−^^3^
0 restraints	Δρ_min_ = −0.30 e Å^−^^3^

### Special details

**Table:**

Experimental. Absorption correction: CrysAlisPro, Agilent Technologies, Version 1.171.35.19 (release 27-10-2011 CrysAlis171 .NET) (compiled Oct 27 2011,15:02:11) Empirical absorption correction using spherical harmonics, implemented in SCALE3 ABSPACK scaling algorithm.
Geometry. All esds (except the esd in the dihedral angle between two l.s. planes) are estimated using the full covariance matrix. The cell esds are taken into account individually in the estimation of esds in distances, angles and torsion angles; correlations between esds in cell parameters are only used when they are defined by crystal symmetry. An approximate (isotropic) treatment of cell esds is used for estimating esds involving l.s. planes.
Refinement. Refinement of F^2^ against ALL reflections. The weighted R-factor wR and goodness of fit S are based on F^2^, conventional R-factors R are based on F, with F set to zero for negative F^2^. The threshold expression of F^2^ > 2sigma(F^2^) is used only for calculating R-factors(gt) etc. and is not relevant to the choice of reflections for refinement. R-factors based on F^2^ are statistically about twice as large as those based on F, and R- factors based on ALL data will be even larger.

### Fractional atomic coordinates and isotropic or equivalent isotropic displacement parameters (Å^2^)

**Table:**

	*x*	*y*	*z*	*U*_iso_*/*U*_eq_	
S1	0.83335 (6)	0.21962 (3)	0.00603 (5)	0.05163 (19)	
Cl1	0.57019 (7)	0.94286 (4)	−0.18264 (9)	0.0821 (2)	
N2	0.81521 (19)	0.39415 (10)	−0.11515 (19)	0.0491 (4)	
N1	0.77525 (18)	0.48923 (10)	−0.10634 (18)	0.0476 (4)	
C9	0.8022 (2)	0.33823 (12)	0.0125 (2)	0.0432 (4)	
N3	0.7653 (2)	0.38062 (13)	0.1416 (2)	0.0605 (5)	
C7	0.8045 (2)	0.54568 (12)	−0.2186 (2)	0.0438 (4)	
C5	0.8203 (2)	0.72184 (13)	−0.2668 (3)	0.0515 (5)	
C4	0.7665 (2)	0.81370 (14)	−0.2571 (3)	0.0565 (5)	
C6	0.74834 (19)	0.64447 (12)	−0.2095 (2)	0.0424 (4)	
C3	0.6403 (2)	0.82766 (12)	−0.1920 (2)	0.0520 (5)	
C1	0.6206 (2)	0.66152 (13)	−0.1440 (3)	0.0529 (5)	
C2	0.5654 (2)	0.75293 (15)	−0.1350 (3)	0.0587 (5)	
C8	0.8862 (3)	0.51830 (16)	−0.3537 (3)	0.0637 (6)	
H5	0.908 (3)	0.7122 (16)	−0.314 (3)	0.065 (7)*	
H4	0.812 (3)	0.8684 (18)	−0.300 (3)	0.068 (7)*	
H1	0.572 (2)	0.6117 (16)	−0.111 (3)	0.053 (6)*	
H2	0.482 (3)	0.7642 (18)	−0.088 (3)	0.069 (7)*	
H9	0.825 (3)	0.3660 (17)	−0.208 (3)	0.068 (7)*	
H10B	0.764 (2)	0.3508 (19)	0.228 (3)	0.064 (7)*	
H10A	0.748 (3)	0.4403 (19)	0.138 (3)	0.061 (6)*	
H24	0.958 (3)	0.477 (2)	−0.323 (3)	0.085 (8)*	
H25	0.913 (4)	0.570 (3)	−0.406 (4)	0.105 (10)*	
H23	0.820 (4)	0.488 (3)	−0.430 (4)	0.115 (12)*	

### Atomic displacement parameters (Å^2^)

**Table:**

	*U*^11^	*U*^22^	*U*^33^	*U*^12^	*U*^13^	*U*^23^
S1	0.0813 (4)	0.0306 (3)	0.0452 (3)	0.00960 (19)	0.0166 (2)	0.00294 (15)
Cl1	0.0895 (5)	0.0360 (3)	0.1238 (6)	0.0149 (2)	0.0247 (4)	0.0101 (3)
N2	0.0750 (11)	0.0292 (7)	0.0452 (8)	0.0056 (7)	0.0163 (7)	0.0029 (6)
N1	0.0665 (10)	0.0281 (7)	0.0493 (8)	0.0045 (6)	0.0121 (7)	0.0020 (6)
C9	0.0566 (10)	0.0317 (8)	0.0422 (8)	0.0027 (7)	0.0100 (7)	0.0003 (6)
N3	0.1046 (15)	0.0321 (9)	0.0482 (9)	0.0092 (8)	0.0234 (9)	0.0027 (7)
C7	0.0549 (10)	0.0308 (8)	0.0459 (9)	−0.0027 (7)	0.0078 (7)	0.0015 (7)
C5	0.0563 (11)	0.0365 (9)	0.0636 (12)	−0.0035 (8)	0.0149 (9)	0.0039 (8)
C4	0.0660 (12)	0.0306 (9)	0.0740 (13)	−0.0058 (8)	0.0130 (10)	0.0066 (9)
C6	0.0532 (10)	0.0304 (8)	0.0434 (8)	−0.0021 (7)	0.0051 (7)	0.0026 (6)
C3	0.0620 (12)	0.0308 (9)	0.0622 (11)	0.0036 (8)	0.0041 (9)	0.0034 (8)
C1	0.0624 (12)	0.0332 (9)	0.0659 (12)	−0.0032 (8)	0.0188 (10)	0.0078 (8)
C2	0.0622 (13)	0.0427 (11)	0.0743 (13)	0.0036 (9)	0.0203 (11)	0.0046 (9)
C8	0.0942 (18)	0.0367 (11)	0.0664 (13)	0.0043 (11)	0.0346 (13)	0.0046 (10)

### Geometric parameters (Å, º)

**Table:**

S1—C9	1.6875 (17)	C5—C6	1.390 (3)
Cl1—C3	1.7443 (18)	C5—H5	0.96 (2)
N2—C9	1.345 (2)	C4—C3	1.369 (3)
N2—N1	1.386 (2)	C4—H4	0.97 (2)
N2—H9	0.89 (3)	C6—C1	1.390 (3)
N1—C7	1.286 (2)	C3—C2	1.375 (3)
C9—N3	1.319 (2)	C1—C2	1.384 (3)
N3—H10B	0.84 (3)	C1—H1	0.89 (2)
N3—H10A	0.85 (3)	C2—H2	0.93 (3)
C7—C6	1.483 (2)	C8—H24	0.90 (3)
C7—C8	1.493 (3)	C8—H25	0.90 (4)
C5—C4	1.386 (3)	C8—H23	0.93 (4)
			
C9—N2—N1	117.64 (15)	C5—C6—C1	118.38 (17)
C9—N2—H9	118.2 (16)	C5—C6—C7	121.38 (17)
N1—N2—H9	122.1 (16)	C1—C6—C7	120.23 (16)
C7—N1—N2	117.88 (15)	C4—C3—C2	121.79 (18)
N3—C9—N2	116.91 (16)	C4—C3—Cl1	119.54 (15)
N3—C9—S1	122.19 (14)	C2—C3—Cl1	118.67 (17)
N2—C9—S1	120.89 (13)	C2—C1—C6	121.34 (18)
C9—N3—H10B	121.6 (17)	C2—C1—H1	120.2 (14)
C9—N3—H10A	118.9 (16)	C6—C1—H1	118.5 (14)
H10B—N3—H10A	119 (2)	C3—C2—C1	118.5 (2)
N1—C7—C6	115.22 (16)	C3—C2—H2	120.4 (16)
N1—C7—C8	125.12 (17)	C1—C2—H2	121.0 (16)
C6—C7—C8	119.66 (16)	C7—C8—H24	112.5 (18)
C4—C5—C6	120.66 (19)	C7—C8—H25	111 (2)
C4—C5—H5	119.0 (14)	H24—C8—H25	115 (3)
C6—C5—H5	120.3 (14)	C7—C8—H23	107 (2)
C3—C4—C5	119.27 (18)	H24—C8—H23	107 (3)
C3—C4—H4	118.2 (15)	H25—C8—H23	103 (3)
C5—C4—H4	122.5 (15)		
			
C9—N2—N1—C7	171.36 (17)	N1—C7—C6—C1	−30.9 (2)
N1—N2—C9—N3	−6.1 (3)	C8—C7—C6—C1	148.1 (2)
N1—N2—C9—S1	174.28 (14)	C5—C4—C3—C2	0.3 (3)
N2—N1—C7—C6	175.21 (15)	C5—C4—C3—Cl1	−178.89 (17)
N2—N1—C7—C8	−3.8 (3)	C5—C6—C1—C2	−0.2 (3)
C6—C5—C4—C3	−0.8 (3)	C7—C6—C1—C2	−179.58 (19)
C4—C5—C6—C1	0.7 (3)	C4—C3—C2—C1	0.2 (3)
C4—C5—C6—C7	−179.92 (18)	Cl1—C3—C2—C1	179.40 (17)
N1—C7—C6—C5	149.71 (19)	C6—C1—C2—C3	−0.3 (3)
C8—C7—C6—C5	−31.3 (3)		

### Hydrogen-bond geometry (Å, º)

**Table:**

*D*—H···*A*	*D*—H	H···*A*	*D*···*A*	*D*—H···*A*
N2—H9···S1^i^	0.89 (3)	2.69 (3)	3.581 (2)	175 (2)
N3—H10*B*···S1^ii^	0.84 (3)	2.54 (3)	3.351 (2)	163 (2)
N3—H10*A*···Cl1^iii^	0.85 (3)	2.88 (2)	3.500 (2)	131 (2)

Symmetry codes: (i) *x*, −*y*+1/2, *z*−1/2; (ii) *x*, −*y*+1/2, *z*+1/2; (iii) *x*, −*y*+3/2, *z*+1/2.
